# Supplementary material for: Systemic immune inflammation index as a predictor of disease severity in tetanus patients: A retrospective observational study
Source: PLoS One. 2024 Dec 31;19(12):e0316196. doi: 10.1371/journal.pone.0316196 (PMC11687931; doi:10.1371/journal.pone.0316196)
Supplement: Attachment — Submitted filename: tetanus manuscript..doc [file pone.0316196.s001.doc]

**Systemic Immune Inflammation Index as a Predictor of Disease Severity in Tetanus Patients: A Retrospective Observational Study**

Dai Cheng^1*^ Ding Wenying^2*^ Huang Jizheng^2#^ Sun Wei^1^ Li Liang^1^ Han Guolei ^1^ Yang Hao^1^

1 Department of Intensive Care Medicine, No.2 People's Hospital of Fuyang City, Fuyang, China

2 Department of Hospital Infection Management, No.2 People's Hospital of Fuyang City, Fuyang, China

* Co-first authors

\# Corresponding author: Huang Jizheng, MPH, hjzh_ly@126.com

**Abstract: Objective:** This study aimed to analyze the predictive value of the systemic immune inflammation index (SII) for the severity of disease in tetanus patients. **Methods**: Clinical data of 34 tetanus patients admitted to the Second People's Hospital of Fuyang from **January 1, 2019 to December 31, 2022** were analyzed. Based on whether patients received intensive care unit (ICU) treatment after admission, the patients were divided into ICU and non-ICU groups. The diagnostic value of SII for the severity of tetanus was assessed. **Results**: Among the 34 patients, 18 (52.90%) were classified into the ICU group, and 16 (47.10%) into the non-ICU group. There were statistically significant differences in white blood cell count, platelet count, neutrophil count, and SII between the two groups (P<0.05). Logistic regression analysis revealed that SII was a risk factor for tetanus patients requiring ICU treatment. The area under the curve (AUC) for SII predicting ICU treatment in tetanus patients was 0.896 (95% CI 0.790–1.000, P<0.001). **Conclusion**: The SII can serve as an objective predictive indicator for tetanus patients requiring ICU treatment.

**Keywords**: Tetanus; Severity; Risk factors

Tetanus is an infectious disease caused by Clostridium tetani, characterized by persistent, tonic contractions of skeletal muscles and paroxysmal spasms in patients[1-2]. Due to the nonspecific early clinical symptoms of tetanus, patients are typically initially admitted to general wards, and transfer to the intensive care unit (ICU) for treatment occurs when the condition becomes severe, leading to delayed intervention and posing a threat to patients' lives. Therefore, guidelines emphasize the need for more graded schemes and clinically relevant studies on prognosis[2-3].

The Systemic Immune Inflammation Index (SII) is a novel immunoinflammatory marker that integrates peripheral blood platelet, neutrophil, and lymphocyte counts. It is considered an effective indicator reflecting the systemic immune-inflammatory status and prognosis, with the formula: Neutrophil count (×10^9^/L) × Platelet count (×10^9^/L) / Lymphocyte count (×10^9^/L)[4]. Studies have shown that SII has excellent clinical value in assessing the prognosis of patients with rheumatoid arthritis, cancer, infectious diseases, and other conditions[5-8]. This study retrospectively analyzed the clinical treatment of 34 tetanus patients, exploring the clinical value of SII in predicting the severity of disease in tetanus patients.

**1 Materials and Methods**

1.1 Study design and patients

The study was approved by the Ethics Committee of No.2 People's Hospital of Fuyang City for the analysis of the data from the database and conducted in accordance with the Helsinki Declaration of 1975. The diagnosis of tetanus conformed to the WHO definition of tetanus.

We reviewed the hospital database **between December 5th, 2023 and December 31st, 2023** that involved adult tetanus patients who were admitted to the No.2 People's Hospital of Fuyang City between **January 1，2019 to December 31，2022.**Inclusion criteria were as follows: 1) Patients clinically diagnosed with tetanus; 2) Complete clinical data. Exclusion criteria were: 1)Discharge or death within 24 hours of admission; 2) Incomplete clinical data. A total of 34 patients were included, comprising 22 males (64.70%) and 12 females (35.30%), with an average age of (62.06±12.88) years.

1.2 Data Collection

Based on whether patients received intensive care unit (ICU) treatment after admission, the 34 patients were grouped into ICU and non-ICU patients. Data recorded included patient gender, age, underlying diseases, Ablett classification, time from onset to medical treatment, total hospital stay, white blood cell count, platelet count, total bilirubin, creatinine, creatine kinase, creatine kinase isoenzyme, neutrophil count, lymphocyte count, and the Systemic Immune Inflammation Index (SII) calculated based on blood routine results.

1.3 Statistical Analysis

Statistical analysis was performed using SPSS 22.0. Continuous data were expressed as "$\overline{x}$±s" and analyzed using independent sample t-tests. Categorical data were presented as case numbers (n) and percentages (%) and analyzed using χ² tests. Binary logistic regression analysis was used for multifactor analysis. Receiver Operating Characteristic (ROC) curves were drawn to analyze the diagnostic value of SII for the severity of tetanus, with the cutoff value determined as the point with the maximum Youden index. A *p* value <0.05 was

considered to be statistically significant.

**2 Results**

2.1 Univariate Analysis of Tetanus Patients Receiving ICU Treatment

Among the 34 patients, 18 (52.90%) were categorized into the ICU group, and 16 (47.10%) into the non-ICU group based on whether they received ICU treatment. There were no statistically significant differences between the two groups in terms of age, gender, underlying diseases, time from onset to medical treatment, total bilirubin, creatinine, creatine kinase, creatine kinase isoenzyme, and lymphocyte count (P>0.05). However, statistically significant differences were observed in white blood cell count, platelet count, neutrophil count, and the Systemic Immune Inflammation Index (SII) between the two groups (P<0.05). Refer to Table 1 for details.

Table 1: Univariate Analysis of Tetanus Patients Receiving ICU Treatment

| Variable | | ICU Group（n=18） |  | Non-ICU Group（n=16） | χ^2^/ *t Value* | *P Value* |
| --- | --- | --- | --- | --- | --- | --- |
| Gender［n(%)］ |  |  |  |  | 0.216 | 0.642 |
|  | Male | 11（61.11） |  | 11（68.75） |  |  |
|  | Female | 7（38.89） |  | 5（31.25） |  |  |
| Underlying Diseases［n(%)］ |  |  |  |  | 2.862 | 0.091 |
|  | Present | 4（22.22） |  | 8（50.00） |  |  |
|  | Absent | 14（77.78） |  | 8（50.00） |  |  |
| Age (years) | | 62.22±12.55 |  | 61.88±13.65 | 0.077 | 0.939 |
| Time from Onset to Treatment (days) | | 2.78±1.66 |  | 3.88±3.59 | -1.164 | 0.253 |
| White Blood Cell Count（×10^9^∕L) | | 10.00±3.14 |  | 6.55±1.99 | **3.776** | **0.001** |
| Platelet Count（×10^9^∕L) | | 284.28±81.61 |  | 219.13±68.19 | **2.508** | **0.017** |
| Total Bilirubin（×10^9^∕L) | | 15.33±5.36 |  | 16.37±9.54 | -0.398 | 0.693 |
| Creatinine（×10^9^∕L) | | 62.39±11.88 |  | 63.94±11.11 | -0.393 | 0.698 |
| Creatine Kinase（×10^9^∕L) | | 334.33±205.73 |  | 222.75±207.19 | 1.573 | 0.125 |
| Creatine Kinase Isoenzyme（×10^9^∕L) | | 21.11±10.77 |  | 18.50±16.33 | 0.556 | 0.582 |
| Neutrophil Count  （×10^9^∕L) | | 8.16±3.36 |  | 4.27±1.84 | **4.114** | **<0.001** |
| Lymphocyte Count  （×10^9^∕L) | | 1.45±0.78 |  | 1.61±0.58 | -0.649 | 0.521 |
| SII | | 2222.15±2197.33 |  | 624.84±349.71 | **3.041** | **0.007** |

2.2 Multivariate Analysis of Tetanus Patients Receiving ICU Treatment

Building upon the results of univariate analysis, variables with statistically significant differences, including white blood cell count, platelet count, neutrophil count, and the Systemic Immune Inflammation Index (SII), were selected as independent variables. Binary logistic regression analysis was conducted with the acceptance of ICU treatment as the dependent variable. The results indicated that SII is a risk factor for tetanus patients requiring ICU treatment. Refer to Table 2 for details.

Table 2: Multivariate Analysis of Tetanus Patients Receiving ICU Treatment

| Variable | β | Wald | *P* Value | OR | 95% C.I.for OR | |
| --- | --- | --- | --- | --- | --- | --- |
|  |  |  |  |  | Lower | Upper |
| SII | 0.003 | 4.029 | 0.045 | 1.003 | 1.000 | 1.006 |
| Neutrophil Count | 0.673 | 3.399 | 0.065 | 1.961 | 0.958 | 4.010 |
| Constant | -6.844 | 6.282 | 0.012 | 0.001 |  |  |

2.3 Predictive Value of SII for Tetanus Patients Receiving ICU Treatment

The SII demonstrated significant predictive value for tetanus patients requiring ICU treatment, with an area under the curve (AUC) of 0.896 (95% CI 0.790–1.000, P<0.001). At the optimal cutoff value of 892.98, the sensitivity of SII in predicting tetanus patients requiring ICU treatment was 83.3%, with a specificity of 87.5%. Refer to Figure 1 for the ROC curve illustrating the predictive performance of SII.

Figure 1: ROC Curve of SII for Predicting ICU Treatment in Tetanus Patients

2.4 Treatment

34 patients with tetanus were all treated with antibiotics, using metronidazole and penicillin. 72.22% (13/18) of the ICU group received TAT treatment, while 56.25% (9/16) of the non-ICU group received TAT treatment. There was no statistically significant difference between the two groups（χ^2^=0.946，*P*=0.331）。100.00% (18/18) of ICU group patients used spasm control drugs, while 68.75% (11/16) of non-ICU group patients used spasm control drugs. The difference between the two groups was statistically significant（χ^2^=0.946，*P*=0.331）。100.00% (18/18) of ICU patients used sedation and mechanical ventilation, while 0% (0/16) of non-ICU patients used sedation and mechanical ventilation, with a statistically significant difference between the two groups（χ^2^=34.000，*P*<0.001）。

Table 3: Comparison of treatment between ICU and non-ICU patients

| Variable | | ICU Group（n=18） |  | Non-ICU Group（n=16） | χ^2^/ *t Value* | *P Value* |
| --- | --- | --- | --- | --- | --- | --- |
| Using TAT | |  |  |  | 0.946 | 0.331 |
|  | Yes | 13 |  | 9 |  |  |
|  | No | 5 |  | 7 |  |  |
| Using spasm control drugs | |  |  |  | － | 0.016^*^ |
|  | Yes | 18 |  | 11 |  |  |
|  | No | 0 |  | 5 |  |  |
| Using sedatives | |  |  |  | 34.000 | <0.001 |
|  | Yes | 18 |  | 0 |  |  |
|  | No | 0 |  | 16 |  |  |
| receiving mechanical ventilation | |  |  |  | 34.000 | <0.001 |
|  | Yes | 18 |  | 0 |  |  |
|  | No | 0 |  | 16 |  |  |

***** **Fisher's exact test method**

2.5 Prognosis

The prognosis of tetanus patients varied based on the initial treatment location. Among those initially treated in the ICU, 6 patients were admitted, 5 were cured, and 1 patient opted for treatment discontinuation. For patients transferred from general wards to the ICU, 12 were admitted, 9 were cured, and 3 patients chose to discontinue treatment. One patient initially admitted to a general ward, who deteriorated but refused transfer to the ICU, opted for treatment discontinuation. All 15 patients continuously treated in general wards were cured. Refer to Table 3 for a comparison of the cure rates among different treatment groups.

Table 4: Comparison of Cure Rates in Different Initial Treatment Settings

| Group | Total Number (cases) | Discontinuation/Death (cases) | Cured (cases) | Cure Rate (%) |
| --- | --- | --- | --- | --- |
| First ICU Treatment | 6 | 1/0 | 5 | 83.33 |
| Transfer to ICU Treatment | 12 | 3/0 | 9 | 75.00 |
| Continuous General Ward Treatment | 15 | 0/0 | 15 | 100.00 |
| Total | 33 | 4/0 | 29 | 87.88 |

**3 Discussion**

Tetanus is a vaccine-preventable disease that remains prevalent in many low- and middle-income countries, posing ongoing challenges[9-10]. Studies suggest that early initiation of intensive care and nursing for severe and very severe (Ⅲ/Ⅳ grade) patients can reduce the mortality rate among tetanus patients[11-12]. Therefore, predicting the severity of tetanus is crucial for patient treatment and prognosis.

The main principles of tetanus treatment include: sedation, analgesia, muscle relaxation to control spasms, correction of autonomic dysfunction to avoid exhaustion; Thorough debridement and anti tetanus clostridium treatment; Neutralizing toxins in the circulatory system; symptomatic and supportive treatment[13]。Some studies have pointed out that airway management is the key to reducing the risk of death. For severe and extra severe patients, early tracheotomy and admission to intensive care units with mechanical ventilation support are recommended for treatment [14]。Therefore, early identification of severe and extra severe patients and early transfer to the ICU for treatment are necessary for the successful treatment of patients.

Currently, the assessment of tetanus severity often relies on the Ablett grading system[2,15]. In this study, 13 patients initially admitted to general wards based on Ablett grading later required transfer to the ICU as their conditions worsened. Some patients exhibited mild symptoms upon admission and deteriorated after being admitted to general wards, leading to delayed ICU treatment. Cai Miaotian et al.[16] highlighted the limitations of the Ablett grading system, emphasizing its subjective nature and lack of quantitative precision. In contrast, the Systemic Immune Inflammation Index (SII) can predict the severity of the disease upon admission, guiding ICU admission and allowing for early intervention to prevent progression to severe conditions. In this study, the AUC of the ROC curve for SII predicting ICU treatment in tetanus patients was 0.896, demonstrating high sensitivity and specificity.

In summary, the SII is a convenient and cost-effective indicator that can serve as an objective predictor for tetanus patients requiring ICU treatment. However, due to the small sample size in this study, further confirmation through larger-scale research is warranted. Additionally, the lack of dynamic monitoring of SII changes during hospitalization and the absence of an in-depth exploration of the correlation between SII changes and the severity of the patient's condition require further investigation in future studies.

**Data Availability** **Statement**

The data that support the findings of this study are available from the corresponding author, upon reasonable request.

**Ethics Statement**

The studies involving human participants were reviewed and approved by Ethics Committee of No.2 People's Hospital of Fuyang City(20231210056).The patients/participants provided their written informed consent to participate in this study. All methods were implemented in accordance with relevant guidelines and regulations.

**References:**

[1] Yen LM, Thwaites CL.Tetanus[J].*Lancet*.2019;393(10181):1657-1668. doi:10.1016/S0140-6736(18)33131-3

[2] Wang CL, Liu S, Chen QJ, et.al. [Specifications for diagnosis and treatment of non-neonatal tetanus[J]. Zhonghua Liu Xing Bing Xue Za Zhi. 2020 Feb 10;41(2):162-166. Chinese. doi: 10.3760/cma.j.issn.0254-6450.2020.02.005.

[3] WEI Jin-gang，WANG Su-xing，QIN Hao. Epidemiological characteristics and deathrisk analysis of severe tetanus in a tertiary hospital，2008－2018 [J]. Pract Prev Med,2020,27(01):50-53.

[4] Hu B, Yang XR, Xu Y,et.al. Systemic immune-inflammation index predicts prognosis of patients after curative resection for hepatocellular carcinoma[J]. Clin Cancer Res,20(23):6212-22. doi: 10.1158/1078-0432.CCR-14-0442.

[5] Liu, B, Wang, J, Li, Y. Y.et al. The association between systemic immune-inflammation index and rheumatoid arthritis: evidence from NHANES 1999-2018[J]. Arthritis research & therapy, *25*(1), 34.DOI:10.1186/s13075-023-03018-6

[6] LI Pei-hang，LU Jia-li，YUE Jin-bo.Relationship between platelet-to- lymphocyte ratioand systemic immune inflammation index and prognostic outcomes in patients with advanced renal cell carcinoma treated with immune checkpoint inhibitors [J]. Chin J Cancer Prev Treat,2022,29(07):523-530.DOI:10.16073/j.cnki.cjcpt.2022.07.12.

[7] Cui Z, Kuang S, Yang X, et al. Predictive value of the systemic immune inflammation (SII) index for stroke-associated pneumonia[J]. Brain Behav. 2023 Dec;13(12):e3302. doi: 10.1002/brb3.3302.

[8] Muhammad S, Fischer I, Naderi S,et al. Systemic Inflammatory Index Is a Novel Predictor of Intubation Requirement and Mortality after SARS-CoV-2 Infection. Pathogens[J]. 2021,10(1):58. doi: 10.3390/pathogens10010058.

[9] Chalya PL, Mabula JB, Dass RM, et al. Ten-year experiences with Tetanus at a Tertiary hospital in Northwestern Tanzania: A retrospective review of 102 cases[J].World Journal of Emergency Surgery, 2011, 6(1):20-27.

[10] [Nóbrega](https://pubmed.ncbi.nlm.nih.gov/?term=Nóbrega+MV&cauthor_id=27478080) MV , [Reis](https://pubmed.ncbi.nlm.nih.gov/?term=Reis+RC&cauthor_id=27478080) RC, Oluk AI ,et al .Patients with severe accidental tetanus admitted to an intensive care unit in Northeastern Brazil: clinical-epidemiological profile andrisk factors for mortality [J].Braz J Infect Dis,2016;20(5):457-461

[11] Fan Z, Zhao Y, Wang S, et al. Clinical features and outcomes of tetanus:a retrospective study[J].Infect Drug Resist, 2019,12:1289-1293.

[12] Mahieu R, Reydel T, Maamar A, et al. Admission of tetanus patients to the ICU:a retrospective multicentre study[J]. Ann. Intensive Care, 2017, 7(1):112-118.

[13]Rodrigo C, Fernando D, Rajapakse S. Pharmacological management of tetanus: an evidence-based review. Crit Care. 2014,18(2):217.

[14]Lisboa T, Ho YL, Henriques Filho GT, Brauner JS, Valiatti JL, Verdeal JC, Machado FR. Guidelines for the management of accidental tetanus in adult patients. Rev Bras Ter Intensiva. 2011 Dec;23(4):394-409.

[15] ZHANG Xiao meng，WANG Yan hua，WANG Chuan lin．. Diagnosis and treatment of severe adult tetanus [J]. Chi J of Emerg Resusc Disaster Med,2018,13(11):1087-1093. DOI:10.3969/j.issn.1673-6966.2018.11.014.

[16] Cai Miaotian, Liang Lianchun, Li Tongzeng,et.al. Analysis of clinical features and prognostic factors of tetanus [J].BEIJING MEDICAL JOURNAL,2018,40(04):318-322.DOI:10.15932/j.0253-9713.2018.04.008.
