# Supplementary material for: Systemic immune inflammation index as a predictor of disease severity in tetanus patients: A retrospective observational study
Source: PLoS One. 2024 Dec 31;19(12):e0316196. doi: 10.1371/journal.pone.0316196 (PMC11687931; doi:10.1371/journal.pone.0316196)
Supplement: Attachment — Submitted filename: Response to rcademic editor and reviewers.docx [file pone.0316196.s002.docx]

****Dear**** ****Academic Editor and reviewers,****

**Thank you very much for your kindly comments on our manuscript.There is no doubt that these comments are valuable and very helpful for revising and improving our manuscript. In what follows, we would like to answer the questions you mentioned and give detailed account of the changes made to the original manuscript.**

****Academic Editor:****

1. **Comment:What was the total number of tetanus patients seen in the hospital during the study period, and what was the number of those that were excluded due to insufficient data,those excluded on the ground that they were discharged within 24 hours of admission, and those excluded because they were below the study adult age range.**

**Reply:In the results section, corresponding explanations have been added and highlighted in red font.“During the study period, a total of 41 tetanus patients were admitted to the Second People's Hospital of Fuyang. Among them, 6 patients were excluded due to insufficient clinical data, and 1 patient was excluded due to discharge within 24 hours after admission. Finally, 34 tetanus patients were included as the study objects.”**

**2．Comment:The study was conducted in adults; however, the age range of adults was not stated. This should be part of the inclusion criteria.**

**Reply: The article has added inclusion and exclusion criteria for research patients，and highlighted in red font.“Inclusion criteria were as follows: ① Patients clinically diagnosed with tetanus; ② Age>18 years old; ③ Complete clinical data. Exclusion criteria were: ① Discharge or death within 24 hours of admission; ② Incomplete clinical data.”**

**3．Comment:The study's retrospective design, which limits control over selection bias and missing data, should be considered a drawback in addition to those mentioned in the discussion section. The recommendation for a much larger study is in order; however, this preferably should be a prospective study.**

**Reply: In the discussion section, corresponding discussions have been added and highlighted in red font.“However, this study is retrospective in nature, which inherently entails drawbacks such as potential data bias, numerous confounding factors, recall bias, and difficulties in ensuring data quality and completeness. In future studies, we will conduct a multi-center, prospective study with an expanded sample size to validate and refine the findings of this research, thereby enhancing its clinical application value.”**

**4．Comment:The references need to be reviewed; most do not conform to PLOS One reference guideline ([https://journals.plos.org/plosone/s/submission-guidelines](https://c05y1x9s.r.us-east-2.awstrack.me/L0/https:%2F%2Fjournals.plos.org%2Fplosone%2Fs%2Fsubmission-guidelines/1/010f01919610db22-08a73f01-ad92-4414-836a-490fa97e9ff2-000000/EnRa9C00ugGvhN-scpuX76_33VI=174)). Furthermore, I could not find references 6, 15 and 16 from literature search.**

**Reply: 17 references were modified to meet the formatting requirements of the journal.References 5, 15, and 16 have been replaced and the format has been modified.**

**5．****Comment:A majority of the References were more than five years; ideally, at least 60-70% of the References should be less than 5 years post publication. This must be addressed.**

**Reply:** **The references in the article have been revised. 17 references, all published in the past 5 years.**

6. ****Comment:** In the online submission form you indicate that your data is not available for proprietary reasons and have provided a contact point for accessing this data. Please note that your current contact point is a co-author on this manuscript. According to our Data Policy, the contact point must not be an author on the manuscript and must be an institutional contact, ideally not an individual. Please revise your data statement to a non-author institutional point of contact, such as a data access or ethics committee, and send this to us via return email. Please also include contact information for the third party organization, and please include the full citation of where the data can be found.**

**Reply: The author has revised the data declaration and added institutional contact information.“The data that support the findings of this study are not openly available due to reasons of sensitivity and are available from the Ethics Committee of No.2 People's Hospital of Fuyang City（fyhmf@163.com）, upon reasonable request.”**

**7.Comment: Can you please upload an additional copy of your revised manuscript that does not contain any tracked changes or highlighting as your main article file. This will be used in the production process if your manuscript is accepted.**

**Reply:Surely. I will upload an unmarked revisied manuscript.**

**8.Comment:Your ethics statement should only appear in the Methods section of your manuscript. If your ethics statement is written in any section besides the Methods, please delete it from any other section.**

**Reply:Thank you for the academic editor's reminder. The author has placed the ethical statement at the end of the article in the Methods section.**

**9.Comment:Please amend either the abstract on the online submission form (via Edit Submission) or the abstract in the manuscript so that they are identical.**

**Reply:Thank you again for the academic editor's reminder. I will pay attention when resubmitting the paper to ensure that the abstract on the online submission form and the abstract in the manuscript are identical.**

****Reviewer #1:****

1- **Comment:There are few scattered spelling mistakes like the word "conformed" in Page 9 and the word "tracheotomy" in Page 18.**

**Reply: Thank you to the reviewer for their meticulous and conscientious work. The author has checked the entire text and corrected any spelling errors.**

2- **Comment:The author highlighted that the total sample size was 34. However, what was the sample size before the exclusion criteria, in other words, how many patients were admitted with tetanus?**

**Reply:In the results section, corresponding explanations have been added and highlighted in red font.“During the study period, a total of 41 tetanus patients were admitted to the Second People's Hospital of Fuyang. Among them, 6 patients were excluded due to insufficient clinical data, and 1 patient was excluded due to discharge within 24 hours after admission. Finally, 34 tetanus patients were included as the study objects.”**

3- **Comment:The method section seems a bit confusing and not reproducible. The author stated that the patients were categorised into ICU and non-ICU group. The word "categorised" seems inappropriate in this context as patients were not randomised. [This categorisation introduces bias]**

****Reply:**To avoid misunderstandings, the author has made modifications to the sentence in the 1.2 data collection section and highlighted it in red font.“We collected clinical data from 34 tetanus patients and divided them into ICU and non ICU groups based on whether they received ICU treatment.”**

4- ****Comment:**The author need to illustrate when was the SII calculated. Was it calculated upon admission? Also, is there a role of monitoring serial or daily SII to predict progression of severity and/or morbidity?**

****Reply:**（1）The SII is calculated within 24 hours of the patient's admission. This is explained in the 1.2 data collection section and highlighted in red font.（2）Thank you to the reviewer for providing a great research idea. Next, we will continuously monitor the SII of tetanus patients and study its guiding role in patient medical treatment.**

****5-Comment:**Comment:Was the treatment similar to both arms of the study (the ICU and non-ICU groups)?**

****Reply:**The author added a 1.3 treatment section in the 1 Materials and Methods section to introduce the treatment methods for the ICU and non ICU groups.**

****6-Comment:**The discussion section highlights the importance of timely management. However, it does not answer the topic clearly which is the role of SII as this is the cornerstone of this manuscript and their is lack of literature review.**

****Reply:** In the discussion section, corresponding discussions have been added and highlighted in red font.“However, this study is retrospective in nature, which inherently entails drawbacks such as potential data bias, numerous confounding factors, recall bias, and difficulties in ensuring data quality and completeness. In future studies, we will conduct a multi-center, prospective study with an expanded sample size to validate and refine the findings of this research, thereby enhancing its clinical application value.”**

****Reviewer #2:****

**Thank you to Professor Yahya Ali Abdulkareem Abodea for reviewing and guiding this article.The author has made modifications to the article, article format, and reference format to meet the publication requirements of the journal PLOS ONE, and has marked them in red in the manuscript.**

The main revision in the paper and the response to the reviewers' comments are detailedly indicated in the attached file“Responses to comments”,and all changes are also highlighted in the manuscript.

We hope you will find our revised manuscript acceptable for publication.

Yours sincerely,

Huang Jizheng
